# The added value of a face-to-face pan-European course—what makes it worth it?

**DOI:** 10.3389/fmed.2024.1387108

**Published:** 2024-06-06

**Authors:** Robert de Leeuw, Judith A. F. Huirne, Christiano Rositto, Mohammed Mabrouk, Pierre Barri, Marlies Bongers, Andreas Thurkow, Ahmed El-Balat, Nikon Vlahos, Hans Brolmann

**Affiliations:** ^1^Department of Obstetrics and Gynaecology, Amsterdam University Medical Center, Vrije Universiteit Amsterdam, Amsterdam, Netherlands; ^2^Amsterdam Reproduction and Development, Amsterdam, Netherlands; ^3^Casa di Cura Santa Famiglia, Rome, Italy; ^4^Fondazione Policlinico Universitario A. Gemelli IRCCS, Rome, Italy; ^5^University College of London Hospitals (UCLH), London, United Kingdom; ^6^The Cleveland Clinic, London, United Kingdom; ^7^Medisch Centrum Maxima, Eindhoven, Netherlands; ^8^Onze Lieve Vrouwen Gasthuis, Amsterdam, Netherlands; ^9^Spital Uster, Uster, Switzerland; ^10^Universitätsspital Zürich, Zurich, Switzerland; ^11^Athens Medical School, Aretaieion University Hospital, Athina, Greece

**Keywords:** continuous education, European education, intercultural competence, skills education, digital education

## Abstract

**Introduction:**

Over the past decade, digital education has seen widespread adoption, particularly accentuated during the COVID-19 pandemic. The post-COVID era has further emphasized the advantages of digital education in terms of cost, availability, and sustainability. However, concerns regarding the efficacy of digital education, particularly in skills-based learning and the absence of social interaction, have been raised. This paper will look at the added value of international, face-to-face, skills-based courses.

**Method:**

This study evaluates the potential added value of face-to-face international skills courses using the European “Gynecology Experts Training for Upcoming Professionals” (GET-UP) course. Focus group discussions were conducted with participants and faculty members to explore beliefs, attitudes, and perceptions regarding face-to-face learning. Qualitative analysis was performed using thematic analysis to identify domains of added value.

**Results:**

The GET-UP course, conducted over 4 days with a diverse European faculty and participants, highlighted several added-value domains. Themes including diversity, role models, preparation, live interaction, and community emerged from the analysis, emphasizing the significance of face-to-face interaction in enriching the learning experience beyond attaining learning goals.

**Discussion:**

The study underscores the importance of face-to-face interaction in educational settings, offering insights into diverse teaching methods, role modeling opportunities, enhanced preparation, live interactions, and fostering a sense of community. While digital education continues to evolve with interactive features, this study suggests that the inherent pressure and dynamics of face-to-face learning provide unique benefits that may not be easily replicated in digital environments. Future research should investigate and validate these findings further to inform educational practices effectively.

## 1 Introduction

Over the past decade, the education landscape has undergone a profound transformation with the increasing integration of digital technologies (1). This shift has been driven by technological advancements and the need for flexible and accessible learning solutions. Particularly noteworthy is the acceleration of digital education adoption during the COVID-19 pandemic, where widespread school closures necessitated a swift transition to remote learning modalities to ensure the continuity of education (2).

In the wake of the pandemic, we find ourselves in a post-COVID era where digital education has gained further traction, supported by its cost-effectiveness, widespread availability, and environmental sustainability (3). The conveniences afforded by digital platforms, such as anytime, anywhere access to educational resources, have positioned them as indispensable tools in modern medical education. However, amidst the recognition of the benefits of digital education, concerns have been raised regarding its efficacy, particularly in skills-based learning (4).

One of the primary advantages of traditional face-to-face education is the personalized guidance and interaction provided by tutors or instructors, which has been perceived as indispensable, especially in skill acquisition (4). The nuanced feedback, hands-on demonstrations, and individualized support offered in face-to-face settings contribute significantly to the learning experience, fostering deeper understanding and skill mastery. Moreover, the absence of social interaction in most forms of digital education has been identified as a notable drawback, with concerns regarding its impact on student engagement, motivation, and interpersonal skills development (5).

Despite the emphasis on achieving learning goals in educational studies, there has been a noticeable oversight in evaluating the potential additional benefits of educational programs, particularly in fostering social interaction and intercultural competence (6). While learning outcomes remain paramount, the value derived from social interaction, sense of belonging, and cultural exchange in face-to-face settings is increasingly recognized as integral to holistic learning experiences (7).

A potential pitfall of current educational literature is that we forget to evaluate this additional profit, focus on learning goals only, and start providing very effective digital education without realizing what we are missing out on. It is suggested that the added value of face-to-face courses lies in broader goal orientation, sense of belonging, social interaction, and intercultural competence (7). However, despite the recognized benefits of face-to-face education, there remains a gap in understanding its comparative advantages over digitally mediated or locally organized courses.

Understanding the added value of face-to-face education is crucial for several reasons. Firstly, it allows educators and policymakers to make informed decisions about the design and implementation of educational programs, ensuring that they effectively meet learners’ diverse needs and preferences (8). Additionally, gaining insights into face-to-face interaction’s unique benefits enables optimal educational resources and instructional strategies to maximize learning outcomes. Ultimately, by comprehensively evaluating the added value of face-to-face education, we can enhance the quality and efficacy of educational experiences, fostering holistic development and lifelong learning opportunities for learners.

Therefore, we aim to evaluate the potential added value of a face-to-face international skills course. Once a year, a European course for minimally invasive gynecological surgery (the Gynecology Experts Training for Upcoming Professionals or the GET-UP course) is organized. This course is aimed at novice, experienced minimal invasive gynecological surgeons and/or residents. The GET-UP course is a 4-day course characterized by a productive mix of lectures, short interactive sessions (*SIC*) and hands-on training (HOT). Each morning starts with a plenary session of 30 min, followed by a HOT or *SIC* session. The afternoon starts again with a plenary session, followed by a HOT session for those who had a *SIC* session in the morning and the other way around. The course offers a diverse European faculty, the opportunity to network with peers and upcoming professionals, and cooperation between 25 European medical centers. Participants are invited to participate by local representatives of the GETUP course, local advertisement, or the faculty. The GET-UP board considers geographical spread, and each faculty member can subjoin a maximum of two participants. Finally, 100 participants are invited to participate. The faculty consists of European endoscopic experts with a wide variety of experience, fields of expertise and know-how. The course aims to improve the learner’s knowledge, skills, and attitude and enforce collaboration, sharing and endorsing a European community of minimally invasive gynecological surgeons. This study will use the GET-UP course as an example of a face-to-face international skills course to evaluate the potential added value. By examining the nuanced experiences and perceptions of participants and faculty members through qualitative analysis, this study aims to shed light on the multifaceted benefits of face-to-face education and inform educational practices in the evolving digital learning landscape.

## 2 Methodology

A focus group discussion using thematic analysis is well-suited for exploring the added value of face-to-face education (9). It allows for an in-depth exploration of participants’ and faculty members’ beliefs, attitudes, and perceptions, capturing diverse perspectives and experiences (10). Thematic analysis is a well-described and accepted way to facilitate the identification of recurring themes and patterns within the qualitative data, providing rich insights into the multifaceted benefits of face-to-face interaction (11). This approach enables researchers to uncover nuanced understandings of the educational process, including the significance of social interaction, role modeling, and community building, thereby comprehensively addressing the research question’s complexity (12).

### 2.1 Study participants

**Figure 1:**
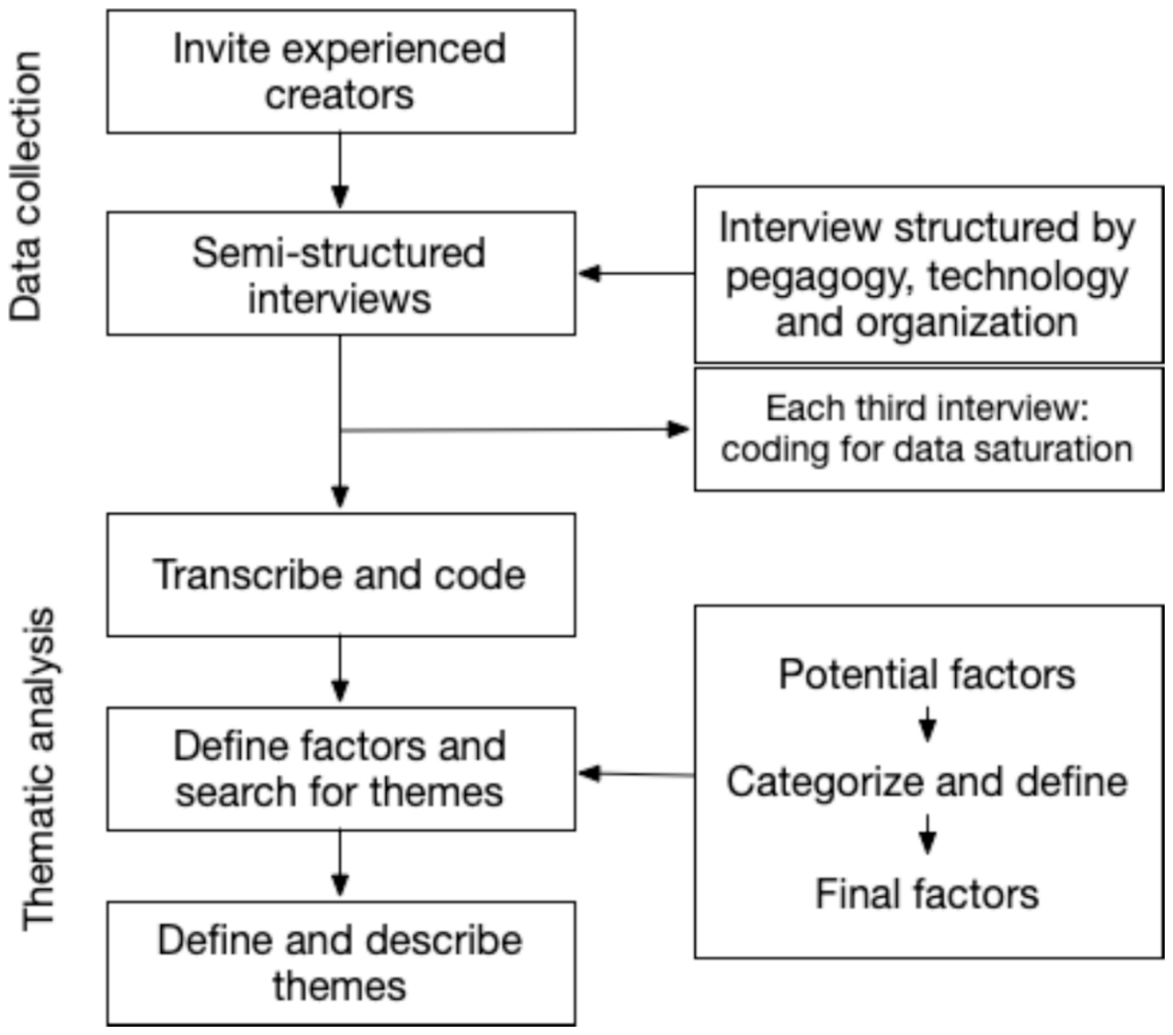
Flowchart of methodology.

We invited both participants and faculty members to a focus group discussion after the course. Before the course started, all participants and faculty were invited to participate in an evaluation. Only participants who attended the whole course and faculty members who were present during all aspects were included to participate. After the course ended, volunteers approached the author RAL for the focus group (Figure 1).

### 2.2 Data collection and analysis

Before the start of the interview, we also provided the participants with a questionnaire containing general demographics and base knowledge. To collect the data, we recorded the focus-group discussions after informed consent from the discussion members. The sessions were facilitated by RAL and lasted between 30 and 45 min. The recordings were anonymously transcribed at verbatim. After transcribing, the interview will be analyzed using Max-QD software, and thematic analysis was used to determine the domains of added value.

To perform the data analysis in a structured method, we used the six steps proposed by Braun et al., containing:

1. Familiarizing oneself with the data,
2. Generating initial codes,
3. Searching for themes,
4. Reviewing themes,
5. Defining and naming themes, and
6. Producing the report (13).

### 2.3 Ethical aspects

All participants were asked to participate before the start of the course and signed an informed consent form. The faculty does not know who refused; therefore, we do not expect a different attitude in the interaction. The Dutch Society for Medical Education gave ethical approval under file number 00833.

## 3 Results

**Figure 2:**
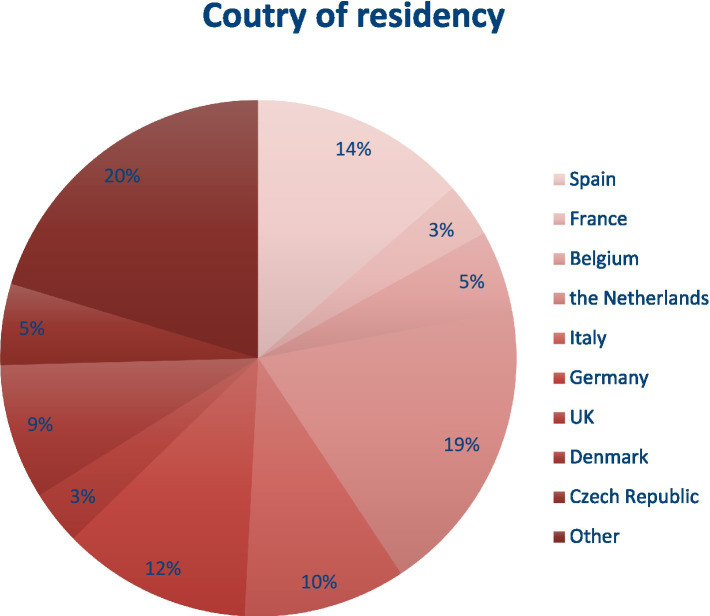
Country of residency of participants.

The GET-UP course took place over 4 days in April 2018. There were 89 participants and 47 faculty members. Of the 59 participants that were included, 83% were in the last year of their residency or just finished, 75% were female, 90% never or rarely had professional contact with a peer from another European country, and 73% never attended another laparoscopy course in their residency (see Figure 2). A total of two interviews were conducted, involving 8 faculty members and 9 participants. Both groups contained participants from Italy, the Netherlands, Great Britain, Denmark, Spain, and Germany and 1 participant came from Romania. 88% of the participants and 25% of the faculty were female.

### 3.1 Reviewing and defining themes

**Figure 3:**
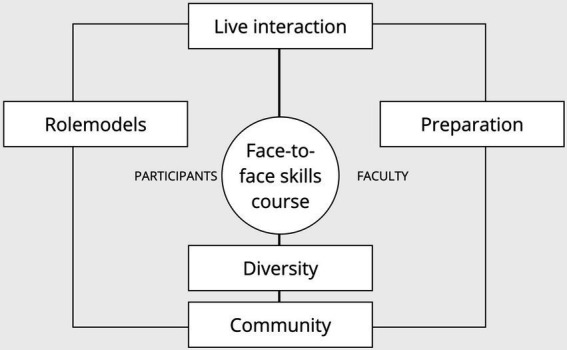
The themes showing the added value of face-to-face learning.

According to the steps of the thematic analysis, all texts were transcribed by RAL to familiarize oneself with the data. In the second step, the transcription was coded into 62 initial codes. Searching within those codes, five themes emerged; diversity, role models, preparation, live interaction, and community (see Figure 3). Reviewing the codes (step 4) did not reveal any other themes. The next step is defining the themes, which will be explained in more detail.

#### 3.1.1 Theme one: Diversity

The first theme that emerged is “Diversity,” which is defined as “a variation in cultural backgrounds, teaching methods, communication skills, and problem-solving methods.” Both faculty and participants highlighted the importance of diversity and the specific added value of this domain to digital education. Several aspects were specifically named as beneficial. The course was international in nature, and participants and faculty from different regional and cultural backgrounds were gathered. This showed the participants the different cultural interpretations of health care and problem-solving.

“*It’s so important to experience different ways of approaching the same problem*” (participant 3).

“*The benefit of mixing with other countries and finding out what’s going on outside of your own country is very valuable*” (participant 5).

The faculty also experienced different aspects of diversity as added value. One important example was diversity in teaching methods, which was discussed by both participants and faculty members. In medicine, teaching is done by each level of experience. Therefore, participants usually also teach back at their clinic, and the participants enjoyed learning about diversity in teaching methods.

“*apart from learning the content of the course, I love to see others teach, so I can adapt my method of teaching*” (participant 2).

#### 3.1.2 Theme two: Role models

The second theme is “Role models,” defined as: “a person showing excellence in craft, teaching abilities and personal qualities.” The participants described the added value of role models during the course. They got inspiration from a person’s excellence in laparoscopic surgery, demonstrated in skills education, videos and presentations. Another aspect was the faculty’s teaching skills, creating inspiration to achieve that level. An important value was learning how to teach. Faculty learned from each other and inspired each other to improve their teaching. Finally, the personal qualities demonstrated in how faculty interact with each other and with the participants allowed participants to get a unique insight into their role models. Participants mentioned that they can be role models for each other. Demonstrating different communication skills and interactions with the faculty, depending on the diverse cultural background.

“*Everybody has a way of working, but we should evaluate this and take the positives from it*” (participant 3).

Finally, participants were inspired by the faculty by showing their vulnerabilities. Saying what they can do, what they cannot do, and where they make mistakes. This showed the participants that their role models are open about their skills and limitations and are willing to get out of their comfort zone. This is illustrated by a quote:

“*It’s very important to get out of your comfort zone, and GET-UP forces you to do so*” (faculty 2).

#### 3.1.3 Theme three: Preparation

The third theme is “Preparation” and is defined as: “the work that has to be done before the course, as part of mandatory lessons, or the creation and/or updating of presentations.” Although the participants enjoyed the mandatory preparation, the faculty mainly stressed the added value of their preparation for the course. The faculty experienced a certain amount of peer pressure between faculty members and the feeling of responsibility for the participants to be more prepared than usual.

“*The dedication and preparation for this course is much higher than to a congress, or even to teaching in the hospital*” (faculty 3).

The possible explanation was that faculty members knew that other faculty members were always present during their presentations and that their sense of community (theme five) pushed them to put in extra effort. They said that when a presentation is due, they usually use an old, existing one. But for this course, they always wanted to do an update just before the course to be up to date. This was also due to the intimate short communications (two or three faculty members and about 10 participants). Here, the faculty felt more responsible for the quality of their teaching than when they were educating without peers or a larger group. The faculty experienced the preparation as the most important part of their own learning process.

#### 3.1.4 Theme four: Live interaction

The fourth theme is called “Live interaction” and is defined as “the communication (verbal and non-verbal) that faculty and participants experience due to their physical presence at the course.” In line with the role models domain, seeing people interact with each other is a very important aspect of a face-to-face course. Both parties said that, apart from learning from short interactions and hands-on training, they learned much from the moments in between. The coffee breaks, the social program in the evening, the pre-and post-course walks and room switching all provided insight into how faculty and participants interacted. The changes in venue, rooms, and assignments (interactive sessions and hands-on training) created an important energy that allowed participants to get more out of the course. This theme overlaps with the role model theme because it addresses communication observation. Yet this was experienced differently because this interaction was about the physical presence of the people themselves. One example is described as:

“*The highest level of training is training*” (faculty 1).

The faculty members said that physical training and being physically together are the highest levels of training. Another example from the participants was that the act of physically moving around and forcing each other to change locations was of great added value:

“*The mix between inactive sessions and hands-on training is very important and keeps everybody fresh and active. I have not fallen asleep once*” (participant 1).

#### 3.1.5 Theme five: Community

Finally, the last theme was “Community,” which is defined as “the feeling of belonging to a group of peers with comparable interests, passions and calling to improve women’s health.” This was one of the most important domains described by participant 2 as:

“*to get other connections from other countries is key for your own development*.”

A feeling of unity was created by the yearly recurrent faculty members, who addressed that seeing each other every year created a sense of community between them. Knowing each other’s expertise, interests, and partially personal life makes a community stick together. This challenging community stimulates critical thinking and stimulates evidence-based care. As faculty 6 said:

“*When you are in a critical, international community, you have to think more evidence-based to support your ideas and treatment*.”

This sense of community helps faculty members contact each other outside of the course and allows easy contact when faced with difficult cases in the clinic. According to the faculty, this is frequently done, and faculty members enjoy the ability to know European experts for advice and refer patients.

“*the collective knowledge is huge*” (faculty 2).

Finally, both faculty and participants enjoyed the networking within the community. Networking allowed new research collaborations, easier consultations with experts and the sharing of contact information for future collaborations.

## 4 Discussion

This study provides five themes that underline the potential additional values of face-to-face learning beyond the initial learning goals. Both faculty and participants provided important insights into aspects rarely evaluated during an educational course. Although diversity in medical education has been frequently addressed in the literature (14), the interpretation of diversity is usually limited to gender and social-cultural aspects. This paper adds unique aspects to the diversity topic, including different teaching styles. Brand et al. write in 2022 that “*Face-to-face meetings facilitate great spontaneity, profound exchange, nuanced communication, personal sharing, and efficient and passionate occurrence of new ideas*,” which this study also supports (15). The importance of role models in the education of residents has been described before by van Delft et al. (2018), who show that subconscious behavior by faculty is experienced by participants and is very difficult to mimic in a digital education environment (16). Brand et al. (2022) write that face-to-face meetings allow one to “*gain wisdom from experienced and devoted leaders*,” which is a variation of the effect of role models (15). Previous studies have also shown the added value of preparing for education (17). Although most studies address this value for students, this paper also shows that faculty can experience great added value in preparing for a course. Faculty members can experience peer pressure to keep their material up-to-date and evidence-based, which increases the chance that they get this added value from preparing for the course. Cullen et al. demonstrated that face-to-face learning during the COVID-19 crisis was still valuable, especially for skills courses (18) and write that live interaction is viable only during face-to-face learning. Enoch et al. write that “*face-to-face learning will always prevail due to the practical skills that doctors need to acquire. The skills that are central to a doctor’s role simply cannot be taught online, and the needs of medical students must be considered in order to produce prepared and competent doctors*” (19).

King et al. compared face-to-face with distance education in 2022 and showed that students found interpersonal interactions an added value of face-to-face learning (20). Michno et al. writes “*students believed that the introduction of online lectures, as a replacement for face-to-face seminars, would have a negative impact on the quality of their medical education*” (21). Another domain from King et al. was the social support network, an important domain in education in general (20). A feeling of community has been described before, and it was found that students were more satisfied. It fostered more meaningful and longitudinal relationships between students and teachers when they experienced a sense of community (22).

The biggest limitation of this study is the focus group members and the sample size. The participants might be biased because they traveled to the course and might want to give socially desirable answers. There is also a selection bias among the participants, where those who did not find benefits from the course did not volunteer for the focus group discussion. The faculty members can be biased toward their opinions as they are part of the face-to-face course. Another limitation is the number of interviews. By analyzing more focus group discussions, other themes might also have appeared. Despite these potential biases and socially desirable answers, this study provides five relevant themes supported by different relevant studies that add value to an international face-to-face skills course. A point can be made that some of these themes might also be possible to address in digital education. More and more digital education is getting interactive; break-out rooms can create a sense of community, and everybody can properly prepare for a digital course. A possible limitation, therefore, lies in the generalizability of the experience in this course, compared to other courses that are provided. Yet this study shows that there is something extra in facing real people. The relative safety of an online environment allows people to hide from their responsibilities, stay hidden inside their comfort zone and prevent exposure to sometimes unwanted but needed social interaction. The underlying pressure of face-to-face education might be something that can and should not always be replaced by a digital variant.

This study is just the beginning of determining the added value of face-to-face learning in a digital world. The themes from this study should be evaluated in different courses with different learning goals. Another step can be determining other domains’ presence in those courses. It is possible that other courses, with different content and learning aims, can provide new insights in other domains. Finally, future studies should evaluate a face-to-face course on the five domains from this study and see how each domain is experienced by the whole group to address their possible generalizability.

## 5 Conclusion

Our study suggests that face-to-face international education may still have a relevant role to play. An in-depth analysis of several domains revealed multiple valuable aspects identified by participants and faculty. While the effort, costs, and sustainability issues related to travel remain concerns, our findings indicate that in an increasingly digital world, there are compelling reasons and benefits for maintaining direct, in-person educational experiences. It is incumbent upon educators to maximize the value of these increasingly vital educational opportunities.”

## Data availability statement

The raw data supporting the conclusions of this article will be made available by the authors, without undue reservation.

## Ethics statement

The studies involving humans were approved by NVMO (Nederlands Vereniging Medisch Onderwijs). The studies were conducted in accordance with the local legislation and institutional requirements. The participants provided their written informed consent to participate in this study.

## Author contributions

RL: Writing – original draft, Writing – review & editing, Conceptualization, Data curation, Formal analysis, Funding acquisition, Investigation, Methodology, Project administration, Resources, Software, Supervision, Validation, Visualization. CR: Conceptualization, Formal analysis, Methodology, Project administration, Validation, Data curation, Investigation, Writing – review & editing. JH: Conceptualization, Formal analysis, Methodology, Project administration, Validation, Supervision, Writing – review & editing. MM: Writing – review & editing. PB: Data curation, Methodology, Supervision, Validation, Writing – review & editing. MB: Conceptualization, Data curation, Formal analysis, Investigation, Methodology, Project administration, Writing – review & editing. AT: Conceptualization, Data curation, Investigation, Methodology, Project administration, Writing – review & editing. AE-B: Conceptualization, Data curation, Investigation, Methodology, Project administration, Validation, Writing – review & editing. NV: Conceptualization, Data curation, Investigation, Methodology, Writing – review & editing. HB: Conceptualization, Data curation, Formal analysis, Investigation, Methodology, Project administration, Supervision, Validation, Writing – review & editing.
